# Unpacking Perceptions on Patient Safety: A Study of Nursing Home Staff in Italy

**DOI:** 10.3390/healthcare12141440

**Published:** 2024-07-19

**Authors:** Ilaria Tocco Tussardi, Stefano Tardivo, Maria Angela Mazzi, Michela Rimondini, Donatella Visentin, Isolde Martina Busch, Emanuele Torri, Francesca Moretti

**Affiliations:** 1 Department of Diagnostics and Public Health, Section of Hygiene, University of Verona, 37134 Verona, Italy; 2 Department of Neurosciences, Biomedicine and Movement Sciences, University of Verona, 37134 Verona, Italy; 3 Department of Prevention, Healthcare Trust of the Autonomous Province of Trento, 38123 Trento, Italy; 4 Clinical Governance Service, Healthcare Trust of the Autonomous Province of Trento, 38123 Trento, Italy

**Keywords:** nursing homes, patient safety culture, Italy, long-term care, healthcare workers, safety perceptions

## Abstract

Nursing homes (NHs) are crucial for de-hospitalization and addressing the needs of non-self-sufficient individuals with complex health issues. This study investigates the patient safety culture (PSC) in NHs within a northern Italian region, focusing on factor influencing overall safety perceptions and their contributions to subjective judgements of safety. A cross-sectional study was conducted on 25 NHs in the Autonomous Province of Trento. The Nursing Home Survey on Patient Safety Culture (NHSPSC) was utilized to assess PSC among NH staff. Multilevel linear regression and post hoc dominance analyses were conducted to investigate variabilities in PSC among staff and NHs and to assess the extent to which PSC dimensions explain overall perceptions of PS. Analysis of 1080 questionnaires (44% response rate) revealed heterogeneity in PSC across dimensions and NHs, with management support, organizational learning, and supervisor expectations significantly influencing overall safety perceptions. Despite some areas of concern, overall safety perceptions were satisfactory. However, the correlation between individual dimensions and overall ratings of safety was moderate, suggesting the need to enhance the maturity level of PSCs. Promoting a shift in PSC could enhance transparency, prioritize resident safety, empower nursing staff, and increase family satisfaction with care provided in NHs. The support provided by management to PSC appears essential to influence NH staff perceptions of PS.

## 1. Introduction

Residential and semi-residential (day-care) facilities play an essential role in caring for the elderly, as they support de-hospitalization and meet the care needs of individuals who are not self-sufficient and/or are affected by complex health issues. In Italy, about 21 in every 1000 elderly individuals reside in nursing homes (NHs), with around 16 out of every 1000 residents being not self-sufficient [1]. Institutionalization rates increase with age, peaking at 76 per 1000 for those over 85 years old [1]. The COVID-19 pandemic has underscored the vulnerability of NHs, where the risk of care errors is notably high, leading to adverse effects on quality of life, morbidity, and mortality [2,3,4,5,6]. The heightened risk stems from various factors, including residents’ multi-morbidity and multiple therapies and the necessity for interdisciplinary coordination, functional dependency, and cognitive impairment, all of which increase the likelihood of serious consequences from errors [2,7]. In addition, the care model of NHs is considerably different from the acute care and outpatient settings, with most of the direct care provided by nurses. Additionally, NHs constitute a real and often permanent living environment for residents, impacting the quality of life and the transmission of diseases [4]. These factors highlight NHs as a care system with unique safety concerns [2].

The promotion of patient safety culture (PSC) has become an internationally recognized priority [8,9], with extensive research conducted in some healthcare settings like hospitals, while NHs have received less attention. Moreover, empirical studies on PSC in NHs predominantly originate from North American contexts, with a scarcity of evidence from European nations, as emphasized by Gartshore et al. in a 2017 scoping review [10]. While some studies on PSC in NHs have been undertaken in Norway [11,12,13], further research is needed to identify barriers to safe care delivery and potential areas for enhancement. It is acknowledged that safety culture varies across countries, necessitating tailored evaluations to devise effective interventions [14,15,16,17].

Measuring PSC remains a contentious issue, given that the core of culture comprises intangible and implicit assumptions [18]. To address this, ‘safety climate’ (the perceived value placed on safety in an organization at a particular time point) has been proposed as a measurable correlate of safety culture, reflecting tangible characteristics through individuals’ attitudes and perceptions [19]. In this article, we will use the term ‘culture’ (the values placed on safety and the extent to which people take personal responsibility for safety in an organization).

In recent years, various tools have been developed to measure PSC, with the Nursing Home Survey on Patient Safety Culture (NHSPSC) being recommended at the European level [20]. This questionnaire, developed by the Agency for Healthcare Research and Quality (AHRQ), has demonstrated good psychometric properties across different countries [21,22,23,24], though its validation in Italian had not been conducted yet.

Despite the usefulness of safety climate questionnaires in pinpointing areas for improvement, safety culture is multidimensional and influenced by staff culture, beliefs, values, and attitudes [25]. Understanding the predictive factors’ interplay and their relative impacts on safety assessments is vital for prioritizing corrective interventions [26]. The study hypothesis is that each dimension of PSC can have a different influence on the formation of the overall judgment and overall perception of safety in the NH. Establishing the actual weight of each dimension in predicting patient safety is crucial for determining priority in implementing corrective interventions.

The aims of this study are:

1. To describe PSC in the NH setting within a northern Italian region;
2. To explore the factors influencing overall safety perceptions and identify their respective contributions to subjective judgments of safety.

## 2. Materials and Methods

### 2.1. Study Design and Setting

This cross-sectional study was conducted on a sample of NH workers in the Autonomous Province of Trento (APT), a region in north-eastern Italy. A single-stage cluster sampling method was used: initially, all NHs were invited to participate, and subsequently, all staff members of participating NHs, including non-clinical professionals such as support and administrative staff, were included in the sampling frame. 

The APT features a unique Healthcare Local Trust responsible for providing care to nearly 550,000 residents both from urban and rural areas. NH care is delivered through the public system, with 55 NHs offering a total of 4600 beds dedicated to non-self-sufficient individuals requiring continuous medical treatment and healthcare assistance not feasible at home [27]. Most residents are elderly. Of these 55 NHs, 25 (45.4%) agreed to participate in this study, forming our cluster sample. The number of beds in these NHs ranged from 38 to 199 (with a mean of 94.7), totaling 2368 beds, of which 207 (9.6%) were private.

The Italian version of the NHSPSC was implemented to investigate PSC among NH staff. Details of the validation process are available in the Appendix A. This study adhered to the Strengthening the Reporting of Observational Studies in Epidemiology (STROBE) guidelines and the user’s guide for the NHSPSC provided by the Agency for Healthcare Research and Quality (AHRQ) [28]. Detailed information on the materials and methods employed in this study has been previously published by our group [29].

The data collection took place between June 2018 and February 2019. Inclusion criteria were being a NH operator. In total, 2478 surveys were distributed and 1224 were returned (49.4%). Detailed information on data collection has been previously published by our group [29]. Staff categories were defined in accordance with the original version of the NHSPSC as follows: Staff Manager (including the administrator, medical director, director of nursing, and physicians due to their low numbers); Administrative Staff (including administrative assistants, admissions staff, billing personnel, secretaries, and human resources staff); Nurses; Direct Care Staff (encompassing nursing assistants/aides, healthcare technicians, and physical therapists); Support Staff (comprising personnel not directly involved in resident care, such as drivers, food service workers, dietary staff, housekeeping staff, laundry staff, and maintenance workers); and Other Providers (such as dietitians, nutritionists, occupational/speech/respiratory therapists, social workers, and psychologists).

### 2.2. Survey Instrument

The original version of the NHSPSC comprises four sections. Section 1 consists of 42 items that assess 12 different patient safety dimensions using a 5-point Likert scale. The Likert scale prompts respondents to indicate their level of agreement with safety statements (ranging from 1 for “Strongly Disagree” to 5 for “Strongly Agree”) or with safety scenarios (ranging from 1 for “Never” to 5 for “Always”). Additionally, respondents have the option to select “Not Applicable/Don’t Know”. Dimension scores were calculated as the mean Likert score across all items within the dimension. The percentage of positive, negative, and missing answers (PPA, PNA, and PMA, respectively) for each survey item and dimension was computed, as described elsewhere [29]. Section 2 aims to provide an overall safety assessment by directly soliciting respondents’ opinions on resident safety using a 5-point scale ranging from “Failing” to “Excellent”. Respondents are also asked, “Would you suggest this NH as safe?” Section 3 comprises seven questions pertaining to respondents’ professional roles in the nursing home. The final section allows respondents to provide personal perspectives on residents’ care and safety [21].

### 2.3. Analyses

Descriptive statistics were used to summarize Likert scores for items and dimensions at both the respondent and NH levels. This dual perspective, which accounts for cluster sampling, assumes that respondents working in the same facility may be more similar to each other than to workers in different facilities since they share the same background/context and common habits. Thus, the research results can focus on differential interpretations, and their implications can be addressed on two distinct levels. Specifically, intraclass correlation (ICC) was computed for each dimension to quantify the impact of NH heterogeneity: values exceeding 0.05 indicate substantial variation between clusters and suggest a multilevel approach, accounting for the nested structure of the dataset. Furthermore, the relationship between NHSPSC dimensions and Overall rating (E2), both within and between NHs, was explored by computing the corresponding Pearson correlation coefficient.

A set of preliminary multilevel linear regressions was applied to select both the respondent features, considered here as covariates, and NHSPSC dimensions (predictors) that significantly influence the overall rating on RS (dependent variable), resulting in a parsimonious model. A post hoc dominance analysis was then employed on the final model to elucidate each predictor’s relative contribution to the dependent variable in terms of the decomposition of the R2 fit index, so predictors accounting for larger proportions of variance were labelled as more important. Specifically, this technique estimated the nested regressions obtained by all possible combinations of the predictors, while the set of covariates was always included; it then calculated the Shapley value decomposition (the average marginal contribution of each predictor across all possible nested models) and ranked each selected predictor [30,31]. 

All analyses were performed using Stata software, version 18 (StataCorp. 2023. Stata Statistical Software: Release 18. College Station, TX, USA: StataCorp LLC.).

## 3. Results

Out of the 1224 received questionnaires, 144 were deemed incomplete or lacked information pertinent to the present study’s outcome and were therefore excluded. Consequently, the analysis encompassed a sample of 1080 questionnaires (44% of those distributed), with response rates ranging from 18% to 82% across the 25 NHs. The characteristics of respondents are detailed in Table 1.

Mean scores for the 12 PSC dimensions are presented in Table 2. The distribution of PPA and PNA for each survey item and dimension can be found in Appendix A (refer to Table A1). The four dimensions with the highest mean scores (i.e., Feedback and Communication about mistakes, Handoffs, Overall Perceptions, and Supervisor Expectations and Actions Promoting Resident Safety, RS) attained mean scores ranging from 3.8 to 4, with PPAs between 68% and 76%. Conversely, the three dimensions with the lowest mean scores (Staffing, Non-punitive response to mistakes, and Management Support for RS) achieved values between 3 and 3.2, with PPAs ranging from 37.7% to 43%. Notably, for these three dimensions, 16 out of 25 NHs (64%), 12 out of 25 (48%), and 11 out of 25 (44%) attained mean scores equal to or less than 3 (further details can be found in Table A3 of the Appendix A).

Regarding the overall safety assessment collected in Section 2, 74.3% of respondents indicated they would tell friends that their NH is safe for their family (E1). The mean value of the overall rating (‘Please give this nursing home an overall rating on resident safety’—E2) was 3.3. Specifically, 42% of respondents rated the level as ‘Very Good/Excellent’, 38% as ‘Good’, and 20% as ‘Fair/Poor’.

The variance in scores between NHs was moderate across all dimensions (ICC range: 0.11–0.20; refer to Table 2), indicating the presence of heterogeneity among facilities. The dimension Management Support for Resident Safety exhibited the highest ICC value (ICC = 0.20), suggesting that NH characteristics can account for 20% of its variability. Overall, the contextual effect was significant (ICC > 0.05; the confidence intervals, in the last column, estimate the presence of a contextual effect in each dimension), supporting the decision to employ a multilevel approach in subsequent analyses. A detailed description of the results of the surveys on PSC, stratified by NH, is provided in Table A3.

**Figure A1:**
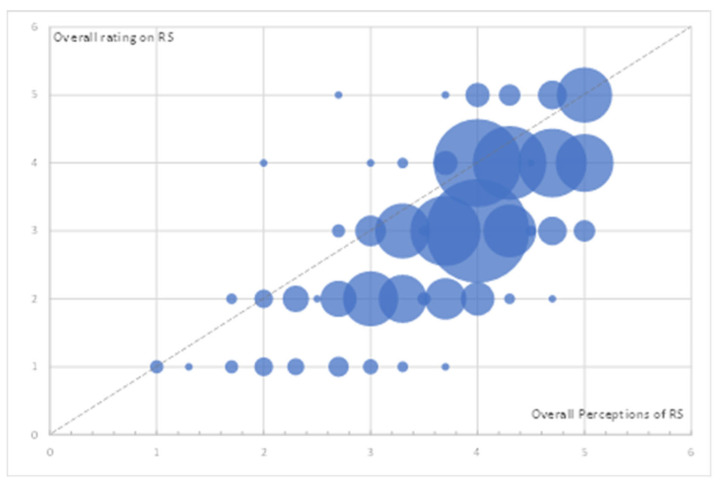
Bubble plot of “Overall rating on resident safety” (E2) against “Overall Perceptions of resident safety” (10th composite dimension): intra-rater comparison. Marker size is weighed by frequencies; the dotted line expresses agreement.

Table 3 presents the results of the exploratory correlation analysis, disaggregated within NHs (above the diagonal) and between NHs (below the diagonal) to differentiate individual- and facility-level correlations. All 12 dimensions and the Overall rating E2 were considered. The between-NH coefficients among the 12 dimensions exhibited high values ranging from 0.61 (between Management Support for RS and Compliance with procedures) to 0.94 (between Supervisor Expectations and Actions Promoting RS and Feedback and Communication); the consistency among these measures supports the multidimensional nature of safety culture. The correlation coefficients between the Overall rating E2 and the 12 dimensions between NH (last row of Table 3) varied between 0.077 and 0.318, indicating only moderate relationships. In the Appendix A, the frequency distribution of Overall rating (E2) and the dimension Overall perceptions of resident safety (Dimension 10) is further explored (Figure A1). The score distribution revealed a tendency towards higher values of the Overall perceptions dimension across all scale points. This result was confirmed with the intra-rater approach with paired data; the value of weighted Cohen’s kappa is 0.31 (95% CI: 0.28–0.34), indicating a low or fair agreement between measures by following Cohen’s suggestions [32].

**Figure 1:**
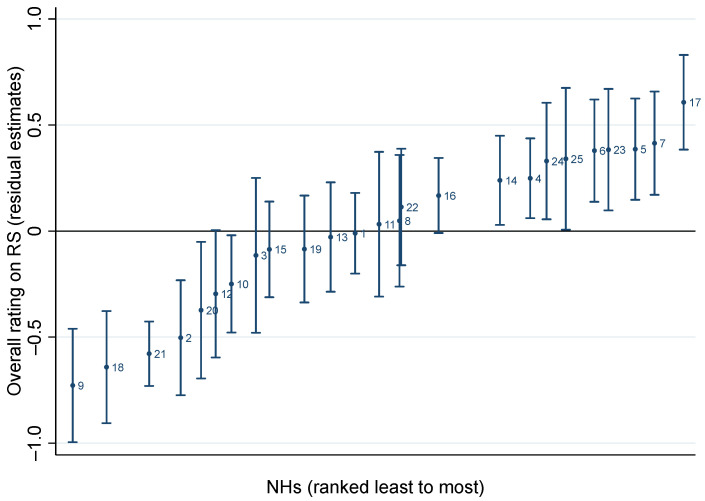
Caterpillar plot of Overall rating on RS, showing NHs residuals and 95% confidence intervals.

Regarding the second aim, Figure 1 illustrates the heterogeneity of the Overall rating among NHs.

Table 4 presents the results of the multilevel final model and dominance analysis: seven dimensions significantly impacted the composition of the overall rating, accounting for the years of work experience of respondents, which emerged as the only covariate. The most influential factors affecting the overall judgment were Organizational Learning, Management Support for RS, and Supervisor expectations and actions promoting RS. The standardized dominance weighs ranged from 0.16 to 0.12, indicating a moderate differential impact.

## 4. Discussion

In this cross-sectional study involving more than 1000 nursing NH providers, we aimed to highlight the safety perspectives of NH staff in Italy. The size of the sample, which represents almost half of all NHs in the study area, along with the satisfactory overall response rate, facilitated a valid portrayal of PSC among NH staff and identified areas for improvement within the NH setting.

The distribution of scores across dimensions exhibited heterogeneity, with a moderate portion of the variation (approximately between 10% and 20%) attributable to the facility level (i.e., affiliation with a specific NH). The observed heterogeneity between NHs suggests the need for strictly shared safety standards able to align expectations regarding safety behaviors. Moreover, the within and between correlations among dimensions suggest that individual factors beyond the facility level may also influence assessments. Indeed, the presence of safety subcultures within institutions is a well-documented phenomenon, although it has not been extensively studied in NHs, especially within the European region [10,11,12,13]. From a recent study conducted by our group, the presence of subcultures in Italian NHs appears to be associated with professional roles, as well as with overarching work-related factors such as seniority, working hours, shifts, and area of activity [29].

To prevent the development of subcultures and achieve successful clinical governance, alignment of leadership with workers is crucial [33,34,35]. Specifically, the support provided to safety culture via management is essential to consistently influence workers’ perception of safety and overall satisfaction [36]. These observations are corroborated in our study by data from the dominance analysis, which emphasized how the three dimensions that most significantly affect overall judgment (Management Support for RS, Organizational Learning, and Supervisor Expectations and Actions Promoting RS) are all related to leadership. 

From the responses to the individual items of Management Support for RS, a perception of management distance from frontline workers emerges, characterized by unsatisfactory receptivity to ideas and suggestions (“Management asks staff how the nursing home can improve resident safety” and “Management listens to staff ideas and suggestions to improve resident safety”) and inadequate implementation of safety walk rounds (“Management often walks around the nursing home to check on resident care”). The high percentage of neutral responses in this dimension (ranging from 16.5% to 39%) supports the perception of hierarchical structures. Moreover, one out of four respondents indicated that management was not actively involved in decisions on how to improve resident safety. As previously noted, managers play a pivotal role in strengthening adaptive capacity within organizations, particularly when they are receptive to new perspectives and foster bottom-up initiatives [13,37]. Involving staff through a bottom-up approach has also been identified as a valuable strategy for ensuring resilient performance in addressing the challenges posed by the COVID-19 pandemic, as evidenced in a study by Ree et al. from 2022 [38]. Furthermore, safety walk rounds are an important and practical tool for enhancing PS within an institution [39]. However, to effectively implement them, it is imperative to proactively cultivate a safety culture to prevent them from being perceived as control measures, particularly in contexts where a punitive culture prevails.

The score for Organizational Learning was not entirely satisfactory, with suboptimal results observed for three out of four items. Specifically, difficulties emerged in implementing changes to improve patient safety (“It is easy to make changes to improve resident safety in this nursing home”). This result somewhat contradicts the positive assessment given to actions taken to improve safety (“This nursing home is always doing things to improve resident safety”), suggesting that despite some proactivity in certain contexts, actions do not seem to yield the perception of change. Ambiguity also arose from the results regarding the ability to learn from adverse events when they occur (“This nursing home lets the same mistakes happen again and again”—negatively worded item). The results on feedback and communication about incidents were satisfactory, indicating that if anything is lacking, it may be the ability to learn from errors.

To enhance the overall perception of safety, management should focus efforts on promoting a climate that facilitates changes and actions for the improvement of safety, as well as monitoring the results of these actions. Additionally, the process of learning from past errors should be promoted from a supervisor/management level so that personnel can perceive that care and attention are allocated to the prevention of adverse events. Organizational learning encourages the dissemination of best practices and evidence-based guidelines throughout the healthcare system, ensuring that lessons learned from past incidents are integrated into future practices. By prioritizing organizational learning, healthcare institutions can proactively mitigate risks, improve care processes, and ultimately enhance patient outcomes, thereby fostering a safer and more reliable healthcare environment [37].

The results concerning Supervisor Expectations and Actions Promoting RS underscore the significance of open communication in bolstering overall safety perceptions. Indeed, all items within the dimension are associated with transparent communication with staff and attentiveness to staff’s work and suggestions. By fostering transparency, trust, collaboration, and shared decision-making, open communication not only mitigates the risk of medical errors but also enhances the overall quality of care [40]. Managers and supervisors must prioritize cultivating a culture of open communication where all stakeholders feel empowered to voice concerns, share information, and collaborate towards the common goal of providing safe and effective care to every patient.

The findings of the descriptive analysis unveiled notable discrepancies in evaluations across dimensions, particularly regarding Staffing, Non-punitive response to mistakes, and the previously discussed Management Support for RS. In fact, nearly half or more than half of the nursing homes recorded scores equal to or below 3 for these dimensions. At the individual level, the three dimensions attained PPAs around 40%, falling well below the satisfactory threshold of 60%, underscoring a pressing need for improvement. Similar outcomes were observed in prior studies [11,12,13] and are consistent with data from the 2019 AHRQ database, which provides benchmarking data from AHRQ survey users [41]. With the exception of Management Support for RS (66% PPA in the AHRQ database compared to 43% in our sample), the dimensions Staffing and Non-punitive response to mistakes exhibited the lowest scores, mirroring trends among the 191 nursing homes included in the AHRQ database. Specifically, Staffing emerged as the most critical area in our sample and demonstrated a comparable average PPA with the AHRQ database (i.e., 37.7% vs. 42%, respectively). For Non-punitive response to mistakes, the disparity between our sample and the reference database was more pronounced (i.e., 38.8% vs. 54%). It is noteworthy that the three dimensions with the highest scores in our sample (feedback and communication about incidents, Supervisor expectations and actions promoting RS, and Overall perceptions of RS) coincided with those scoring highest in the AHRQ database.

Regarding the Staffing dimension, the dominance analysis underscored its significance in shaping the final perception of safety. It is reasonable to assume that insufficient staffing levels and high turnover contribute to heavy workloads and difficulties in ensuring adequate patient safety, as indicated by low scores for items such as “We have enough staff to handle the workload”, “Staff have to hurry because they have too much work to do”, and “It is hard to keep residents safe here because so many staff quit their jobs”. Additionally, the notable percentage of neutral and missing responses for individual items is noteworthy. While these responses may genuinely reflect a lack of clear opinion, they may also signify a reluctance to express negative perspectives. The literature highlights how high turnover is a prevalent issue in long-term care settings [42,43,44]. Turnover rates serve as useful indicators of NH quality and necessitate regular assessment and analysis to identify potential issues and provide necessary improvements [45].

Indeed, evidence suggests that high turnover may result in several adverse consequences for NH residents, such as an increased occurrence of physical restraint [45]. Moreover, it is likely that high turnover rates lead to a greater reliance on shortcuts during procedures, potentially compromising infection prevention and control, as evidenced during the COVID-19 pandemic [46]. Our results partially support this hypothesis, as the two items regarding compliance with procedures (“Staff use shortcuts to get their work done faster” and “To make work easier, staff often ignore procedures”) garnered notable percentages of negative answers (respectively, 1 out of 4 and 1 out of 5 respondents agreed with these statements). Inadequate staffing may also have a detrimental impact on staff well-being, resulting in work overload and burnout [47,48]. Burnout, in turn, can affect both RS and the quality of care, creating a concerning cycle that underscores the importance of monitoring this indicator.

Regarding the Non-punitive response to mistakes dimension, the results highlighted the prevalence of a punitive safety culture among operators. To explain the deviation from the AHRQ database, we hypothesize that this is a particularly critical area in the Italian context. In general, an effective error-response mechanism necessitates that operators be adequately prepared to report mistakes, a responsibility that should be shouldered by management through targeted training and continuous feedback. Providing feedback is a crucial aspect of fostering a positive PSC. A study by Zwijnenberg et al. delved into healthcare professionals’ perspectives on feedback from a PSC assessment [49]. The vast majority (84%) of respondents indicated that feedback partly or wholly stimulated actions to improve PSC, enabling staff to navigate the learning process through the mistakes themselves.

Specifically, regarding the Italian setting, a study by Tereanu et al. explored PSC in Italian territorial prevention facilities in Northern Italy [50]. The Non-punitive response to mistakes dimension scored a 39.5% PPA (38% among nurses and nurse aides) and ranked second lowest after Teamwork across units. The study also compared 10 composite measures with results from hospital settings (Italy and the US), health districts (Spain), and primary healthcare settings (Iran, Turkey). Italian hospitals scored lower (35%) than Italian territorial prevention facilities, which, in turn, scored lower than US hospitals (44%). Additionally, the study sample scored lower than the health district in Spain (42%). Overall, data from Italian settings indicate a generally low and less-developed safety culture in territorial facilities compared to hospitals, characterized by a persistent blame culture and under-reporting of incidents [51].

We also observed that staff expressed the need for more training (“Staff have enough training on how to handle difficult residents”), while simultaneously perceiving difficulties in implementing changes. This indicates the necessity of providing practical training through improvement projects that involve collaboration between staff and management to effectively introduce changes. However, despite the survey results, the dominance analysis indicates that this dimension does not significantly influence the overall perception of safety.

Furthermore, although the scores for some dimensions were not entirely positive, the Overall Perception of Resident Safety (dimension 10) achieved a satisfactory PPA of 76.4%, and the Global assessment section also showed positive scores. Moreover, the correlation between the twelve dimensions and the Overall rating (E2) was only moderate. These unexpected results suggest that item E2 provides additional information compared to that of individual dimensions, prompting reflections on the process of judgment generation by staff regarding their own NH. When tasked with assessing specific safety aspects, staff seem capable of identifying limitations. However, there appears to be a lack of ability to recognize these limitations as important threats to overall safety. Promoting an appropriate “preoccupation with failure”, an essential element of a high-reliability organization, is crucial for improving safety culture. In this regard, sharing the results of the discrepancy between the scores of individual dimensions and the overall rating can help enhance this awareness.

In terms of actionable strategies that can be planned for implementing improvements, suggestions can be found in a practical guideline for PSC improvement promoted by the English NHS. The tool provides a comprehensive ‘toolkit’ to understand how to craft, create, and nurture a positive safety culture and offers a theoretical foundation for how to shift the culture. Among the key elements supporting a positive PSC are leadership, teamwork, communication, and organizational development [52]. A recent review by Taji et al. indicates that strategies for improving PSC in the hospital setting can be categorized into educational, simulation, team strategies, and comprehensive programs [53]. The review emphasizes that all types of strategies have a positive influence on PSC. Another recent review on strategies for improving PSC conducted by Mistri et al. highlights how education and training of healthcare professionals are crucial for strengthening systems and provides the descriptions of specific actions of improvement [54].

### Strengths and Limitations

This study is part of the first attempt to assess PSC in the NH setting in Italy. It should be noted that this study was conducted on a single Italian region and on a limited number of NHs, and therefore the results may not be fully representative of the entire long-term care setting in Italy. The 25 NHs included in this study constituted a convenience sample, which could introduce potential research bias. The significant variation in the response rate of individual NHs may conceal additional biases related to the specific characteristics of the NH. Data were self-reported and possibly subject to social desirability biases. We have, however, limited the collection of socio-demographic information, which could influence the tendency to provide answers that are considered ‘desirable’.

Additionally, the benchmarking comparison was performed using data from the AHRQ database, which primarily consists of information from North American NHs. Nevertheless, a notable strength of this study lies in the validation of the NHSPSC in Italian, providing a standardized tool for comparisons with other Italian settings, thereby enhancing the utility of benchmarking analyses.

In summary, this study serves as a foundational step for further exploration of PSC in the Italian NH context, through the development of a multi-centric study. Particularly, given the presumed association between PSC and actual safe care, further research is warranted to quantify this association with specific outcomes (such as falls, development of pressure ulcers, medication errors, adverse drug events, unplanned transfers to the hospital, etc.) within the country-specific NH context. Future research developments include the realization of a longitudinal study that could provide better insights into how improvements in management practices and organizational culture can influence PSC over time.

## 5. Conclusions

Measuring the safety culture of an organization is the primary and fundamental step towards instigating change and improvement. Ultimately, a shift in safety culture could cultivate an environment within NHs where transparency is esteemed, residents’ safety is prioritized, nursing staff feel empowered, and families are satisfied with the care provided.

## Figures and Tables

**Table 1:** Background characteristics of responders and NHs.

Responders (n = 1080)	N (%)
Staff category	
Direct Care Staff	675 (65)
Nurse	154 (15)
Other Provider	67 (6)
Staff Manager ^1^	66 (6)
Support Staff	42 (4)
Administrative Staff	32 (3)
Job tenure, number of years	
<1	121 (12)
1–2	122 (12)
3–5	134 (13)
6–10	176 (17)
≥11	479 (46)
missing	48 (4)
Work hours per week, n	
<15	19 (2)
16–24	215 (21)
25–40	760 (73)
>40	49 (5)
Work directly with residents	
Yes	907 (87)
No	133 (13)
Nursing Homes (n = 25)	
Staff size, n	
≤30	6 (24)
31–60	13 (52)
61–90	4 (16)
≥91	2 (8)
Beds, n	
≤60	8 (32)
61–100	10 (40)
101–150	4 (16)
≥151	3 (12)

^1^ Physicians were included among Staff Managers due to their low number (n = 2).

**Table 2:** Descriptive of patient safety culture dimensions and staff evaluations on own NH’s safety (n = 1080).

Dimensions	Mean	SD	NH min	NH max	ICC	95% CI
1. Teamwork within units	3.4	0.8	2.9	4.0	0.12	0.07; 0.22
2. Staffing	3.0	0.7	2.5	3.6	0.11	0.06; 0.19
3. Compliance with Procedures	3.6	0.8	3.1	4.3	0.11	0.06; 0.20
4. Training and Skills	3.5	0.7	2.7	4.3	0.17	0.10; 0.28
5. Non-punitive Response to Mistakes	3.1	0.8	2.5	3.8	0.12	0.06; 0.22
6. Handoffs	3.9	0.8	3.2	4.6	0.16	0.09; 0.27
7. Feedback and Communication about Incidents	4.0	0.8	3.4	4.5	0.13	0.07; 0.23
8. Communication Openness	3.4	0.8	2.9	3.9	0.11	0.06; 0.20
9. Supervisor Expectations and Actions Promoting RS	3.8	0.8	3.2	4.2	0.12	0.06; 0.21
10. Overall Perceptions of RS	3.9	0.7	3.4	4.5	0.17	0.10; 0.28
11. Management Support	3.2	1.0	2.5	3.8	0.20	0.11; 0.31
12. Organizational Learning	3.6	0.7	2.9	4.1	0.18	0.11; 0.29
Global assessment						
E1: Willingness to recommend own NH *	74%	44	36%	95%	0.18	0.10;0.30
E2: Overall rating on RS	3.3	0.9	2.4	3.9	0.17	0.10; 0.28

RS = Resident Safety; NH = Nursing Home. * Answering “yes” to the item: “I would tell friends this nursing home is safe”.

**Table 3:** Relationship between NHSPSC dimensions and Overall rating on RS: within (above diagonal) and between (below diagonal) NHs correlation matrix.

	1.	2	3	4	5	6	7	8	9	10	11	12	E2
1. Teamwork	-	0.444	0.428	0.501	0.526	0.419	0.516	0.533	0.538	0.488	0.514	0.545	0.422
2. Staffing	0.775	-	0.377	0.423	0.453	0.396	0.353	0.413	0.392	0.422	0.443	0.445	0.363
3. Compliance with Procedures	0.832	0.793	-	0.402	0.394	0.283	0.349	0.277	0.35	0.419	0.3	0.412	0.318
4. Training and Skills	0.762	0.724	0.695	-	0.416	0.433	0.463	0.424	0.447	0.499	0.477	0.507	0.411
5. Non-punitive Response to Mistakes	0.822	0.830	0.796	0.857	-	0.415	0.475	0.542	0.469	0.433	0.5	0.522	0.388
6. Handoffs	0.652	0.849	0.737	0.700	0.823	-	0.594	0.554	0.511	0.505	0.467	0.53	0.418
7. Feedback and Communication	0.824	0.784	0.836	0.779	0.871	0.882	-	0.597	0.608	0.552	0.535	0.646	0.438
8. Communication Openness	0.752	0.674	0.628	0.731	0.771	0.789	0.882	-	0.611	0.469	0.59	0.582	0.416
9. Supervisor Expectations and Actions Promoting RS	0.829	0.706	0.834	0.744	0.812	0.806	0.940	0.866	-	0.577	0.579	0.633	0.458
10. Overall Perceptions of RS	0.790	0.787	0.779	0.699	0.710	0.764	0.872	0.809	0.818	-	0.59	0.705	0.633
11. Management Support for RS	0.682	0.644	0.609	0.710	0.650	0.661	0.766	0.794	0.674	0.837	-	0.649	0.49
12. Organizational Learning	0.836	0.821	0.818	0.824	0.824	0.819	0.938	0.835	0.857	0.927	0.846	-	0.541
E2 Overall rating	0.194	−0.11	0.174	0.296	0.108	0.077	0.218	0.318	0.303	0.095	0.148	0.149	-

**Table 4:** Multilevel model of overall rating on resident safety and dominance analysis.

Predictors	B (SE)	Std DW	Ranking
Organizational Learning	0.35 (0.05) **	0.1632	1
Management Support for RS	0.18 (0.03) **	0.1540	2
Supervisor Expectations and Actions Promoting RS	0.06 (0.04) *	0.1538	3
Handoffs	0.10 (0.03) **	0.1458	4
Teamwork	0.07 (0.03) *	0.1337	5
Training and Skills	0.09 (0.04) *	0.1275	6
Staffing	0.07 (0.03) *	0.1180	7
Work years (reference: 1 year+)	0.19 (0.06) **	0.0040	8
Pseudo R^2^—level 1	0.42		
Pseudo R^2^—level 2	0.76		

SE = standard error; * 0.01 < *p*-value < 0.05; ** *p*-value < 0.01; Std DW = Standardized Dominance Weights.

## Data Availability

The data presented in this study are available upon reasonable request to the corresponding author.

## Appendix A

### Validation of the Italian Version of NHSPSC

The Italian version of the NHSPSC was developed following the seven steps outlined in the AHRQ translation guidelines [55]. A dedicated team, consisting of five researchers with fluency in English and diverse expertise, undertook the translation process and made cultural adaptations tailored to the Italian context. Specifically, the team comprised two researchers experienced in safety culture assessment and questionnaire translation (F.M. and I.T.T.), one expert in questionnaire validation methodology (M.A.M.), one with direct care experience in nursing homes (D.V.), and one specializing in accreditation processes (E.T.).

To ensure accuracy, the translated questionnaire was back translated into English for verification. Subsequently, a draft version was pre-tested with a focus group comprising seven NH staff members with varying professional backgrounds, including direct care, administrative, and support roles, to evaluate item comprehensibility, relevance, and clarity. A few suggestions for improvement were provided. The primary modification involved item B3 (“We have all the information we need when residents are transferred from hospital”), which was split into two items (B3a and B3b) based on the patient’s transfer origin: hospitals/other nursing homes (item B3a: “We have all the information we need when residents are transferred from a hospital or another nursing home”) or home (item B3b: “We have all the information we need when residents are transferred from home”). The final version of the questionnaire retained 43 items. Information pertaining to the additional item was integrated into the score of the ‘Handoffs’ dimension, ensuring that the survey’s score range remained unaffected, as mean Likert scores were calculated accordingly.

The reliability of the Italian version showed good values, with Cronbach’s alpha ranging from 0.58 to 0.89 (Table A2). Confirmatory factor analysis was conducted using the structural equation model approach to test how the original 12-factor solution fitted the Italian data. The estimated model showed acceptable fit values: RMSEA upper limit < 0.07 (RMSEA = 0.054, 90% confidence interval: 0.051—0.056); CFI > 0.90 (CFI = 0.912); TLI > 0.90 (TLI = 0.900); SRMR < 0.08 (SRMR = 0.056 relative/normed chi-square ratio—χ2/df—between 2 and 5 (χ2 = 2372.32, df = 794, *p* < 0.01)) [56].

**Table A1:** Frequencies of NHSPSC dimensions and items (n = 1080).

Dimension and Item	PPA (%)	PNA (%)	PMA (%)
**1. Teamwork**	**50.8**	**15.8**	**1.2**
A1. Staff in this nursing home treat each other with respect.	49.3	18.2	1.2
A2. Staff support one another in this nursing home.	55.2	13.0	0.7
A5. Staff feel like they are part of a team.	41.7	22.6	1.9
A9. When someone gets really busy in this nursing home, other staff help out.	56.9	9.3	1.2
**2. Staffing**	**37.7**	**37.7**	**2.8**
A3. We have enough staff to handle the workload.	21.7	52.9	1.3
A8. Staff have to hurry because they have too much work to do. (N)	16.9	58.6	0.8
A16. Residents’ needs are met during shift changes.	70.3	8.9	4.5
A17. It is hard to keep residents safe here because so many staff quit their jobs. (N)	42.0	30.4	4.8
**3. Compliance with Procedures**	**60.2**	**15.9**	**2.7**
A4. Staff follow standard procedures to care for residents.	81.3	5.2	1.4
A6. Staff use shortcuts to get their work done faster. (N)	44.6	24.4	3.4
A14. To make work easier, staff often ignore procedures. (N)	54.6	18.2	3.4
**4. Training and Skills**	**58.9**	**14.9**	**2**
A7. Staff get the training they need in this nursing home.	70.5	9.9	1.1
A11. Staff have enough training on how to handle difficult residents.	45.7	24.4	2.1
A13. Staff understand the training they get in this nursing home.	60.5	10.5	2.8
**5. Non-punitive Response to Mistakes**	**38.8**	**27.1**	**5.3**
A10. Staff are blamed when a resident is harmed. (N)	32.2	26.0	7.5
A12. Staff are afraid to report their mistakes. (N)	39.6	28.3	3.3
A15. Staff are treated fairly when they make mistakes.	41.8	30.3	5.7
A18. Staff feel safe reporting their mistakes.	41.6	23.8	4.6
**6. Handoffs**	**67.5**	**7.9**	**3.4**
B1. Staff are told what they need to know before taking care of a resident for the first time.	69.4	8.3	2.7
B2. Staff are told right away when there is a change in a resident’s care plan.	68.0	6.7	4.1
B3. We have all the information we need when residents are transferred from the hospital.	61.4	10.3	4.5
B3b. We have all the information we need when residents are transferred from their homes.	61.4	10.1	4.4
B10. Staff are given all the information they need to care for residents.	77.4	3.9	1.3
**7. Feedback and Communication about Incidents**	**74.4**	**7.1**	**2.2**
B4. When staff report something that could harm a resident, someone takes care of it.	71.9	8.0	3.3
B5. In this nursing home, we talk about ways to keep incidents from happening again.	70.2	10.1	1.9
B6. Staff tell someone if they see something that might harm a resident.	85.7	2.4	2.0
B8. In this nursing home, we discuss ways to keep residents safe from harm.	69.9	7.9	1.8
**8. Communication Openness**	**47.8**	**18**	**1.7**
B7. Staff ideas and suggestions are valued in this nursing home.	44.3	18.7	0.9
B9. Staff opinions are ignored in this nursing home. (N)	38.8	21.1	2.6
B11. It is easy for staff to speak up about problems in this nursing home.	60.3	14.2	1.6
**9. Supervisor Expectations and Actions Promoting Resident Safety**	**70.3**	**9**	**1.3**
C1. My supervisor listens to staff ideas and suggestions about resident safety.	69.8	7.7	1.0
C2. My supervisor says a good word to staff who follow the right procedures.	59.2	13.6	2.2
C3. My supervisor pays attention to safety problems in this nursing home.	81.9	5.8	0.7
**10. Overall Perceptions of Resident Safety**	**76.4**	**4.9**	**1**
D1. Residents are well cared for in this nursing home.	82.7	2.7	0.6
D6. This nursing home does a good job keeping residents safe.	71.7	6.9	1.4
D8. This nursing home is a safe place for residents.	74.8	5.1	0.9
**11. Management Support for Resident Safety**	**43**	**25.1**	**4.6**
D2. Management asks staff how the nursing home can improve resident safety.	46.3	22.0	4.4
D7. Management listens to staff ideas and suggestions to improve resident safety.	48.3	16.5	3.4
D9. Management often walks around the nursing home to check on resident care.	34.4	36.9	5.9
**12. Organizational Learning**	**57**	**12.8**	**4.2**
D3. This nursing home lets the same mistakes happen again and again. (N)	58.9	14.2	4.5
D4. It is easy to make changes to improve resident safety in this nursing home.	44.2	19.7	3.2
D5. This nursing home is always doing things to improve resident safety.	65.6	8.2	2.3
D10. When this nursing home makes changes to improve resident safety, it checks to see if the changes worked.	59.3	9.0	6.9

Possible Likert score range is 1 to 5 points. Positive responses were considered ‘Agree/Strongly Agree’ or ‘Most of the Time/Always’ (score 4–5 on Likert scale) for positively worded items and, ‘Disagree/Strongly Disagree’ or ‘Rarely/Never’ (score 1–2 on Likert scale) for negatively worded items. The opposite applied when calculating the percentage of negative answers. PPA = percentage of positive answers (Likert 4–5). PNA = percentage of negative answers (Likert 1–2). PMA = percentage of missing answers. (N) = negatively worded items. The dimensions’ scores consisted of the mean PPA, PNA, and PMA considering all items included in the dimension.

**Table A2:** Reliability analysis of the Italian NHSPSC and comparison with referral studies from North America and Europe.

Dimension	Item	Cronbach’s Alpha		
		Italy	U.S. [15]	Norway [23]
1. Teamwork	A1, A2, A5, A9	0.87	0.79–0.83 *	0.79
2. Staffing	A3, A8, A16, A17	0.58	0.62	0.55
3. Compliance with Procedures	A4, A6, A14	0.70	-	0.58
4. Training and Skills	A7, A11, A13	0.68	-	0.67
5. Non-punitive Response to Mistakes	A10, A12, A15, A18	0.72	0.78	0.65
6. Handoffs	B1, B2, B3, B3b, B10	0.89	0.81	0.74
7. Feedback and Communication about Incidents	B4, B5, B6, B8	0.84	0.78	0.74
8. Communication Openness	B7, B9, B11	0.71	0.73	0.74
9. Supervisor Expectations and Actions Promoting Resident Safety	C1, C2, C3	0.84	0.79	0.84
10. Overall Perceptions of Resident Safety	D1, D6, D8	0.83	0.74	0.90 **
11. Management Support for Resident Safety	D2, D7, D9	0.85	0.79	0.90 **
12. Organizational Learning	D3, D4, D5, D10	0.72	0.71	0.90 **

* Teamwork across units: 0.79; teamwork within units: 0.83. ** Management and Organizational learning: the factor includes “overall perception of safety”, “management support for patient safety”, and “organizational learning”.

**Table A3:** Descriptive of patient safety culture dimensions per NHs (SD).

NH	1	2	3	4	5	6	7	8	9	10	11	12
1	3.2 (0.8)	2.9 (0.6)	3.5 (0.7)	3.5 (0.7)	3.1 (0.7)	3.8 (0.7)	3.9 (0.8)	3.4 (0.7)	3.8 (0.8)	3.9 (0.7)	3.2 (0.9)	3.5 (0.6)
2	3.4 (0.9)	2.7 (0.7)	3.6 (0.8)	3.3 (0.7)	3.1 (0.7)	3.9 (0.6)	4.1 (0.8)	3.4 (0.8)	3.9 (0.6)	3.6 (0.7)	2.6 (1.0)	3.4 (0.7)
3	3.2 (0.7)	2.6 (0.5)	3.2 (0.4)	3.2 (0.6)	2.7 (0.4)	3.6 (0.8)	3.8 (0.8)	3.4 (0.8)	3.6 (0.8)	3.8 (0.7)	3.0 (0.9)	3.4 (0.6)
4	3.8 (0.7)	3.1 (0.5)	3.7 (0.6)	3.8 (0.6)	3.2 (0.7)	3.6 (0.7)	4.1 (0.8)	3.6 (0.7)	3.8 (0.7)	4.2 (0.5)	3.6 (0.8)	3.7 (0.6)
5	3.4 (0.7)	3.3 (0.6)	3.6 (0.6)	3.5 (0.7)	3.2 (0.7)	4.3 (0.6)	4.3 (0.7)	3.9 (0.8)	3.9 (0.8)	4.2 (0.5)	3.7 (0.9)	3.8 (0.6)
6	4.0 (0.8)	3.6 (0.8)	4.3 (0.7)	4.3 (0.7)	3.8 (0.9)	4.5 (0.6)	4.5 (0.6)	3.6 (0.7)	4.2 (0.6)	4.4 (0.6)	3.8 (1.0)	4.1 (0.6)
7	3.7 (0.6)	3.1 (0.7)	3.7 (0.6)	3.7 (0.6)	3.3 (0.6)	4.2 (0.6)	4.2 (0.5)	3.7 (0.7)	4.0 (0.6)	4.2 (0.5)	3.7 (0.7)	3.8 (0.5)
8	3.3 (0.5)	3.0 (0.6)	3.3 (0.7)	3.3 (0.6)	2.9 (0.6)	3.9 (0.7)	3.8 (0.7)	3.4 (0.7)	3.6 (0.6)	3.9 (0.5)	2.7 (0.6)	3.3 (0.6)
9	2.9 (0.8)	2.7 (0.6)	3.1 (0.7)	3.2 (0.7)	2.8 (0.8)	3.8 (0.7)	3.5 (0.9)	3.0 (0.7)	3.2 (1.0)	3.4 (0.7)	2.5 (1.0)	2.9 (0.8)
10	3.0 (0.8)	2.8 (0.6)	3.4 (0.8)	3.4 (0.6)	3.0 (0.5)	4.0 (0.9)	3.9 (0.9)	3.2 (0.8)	3.7 (0.8)	3.5 (0.8)	2.5 (0.8)	3.3 (0.8)
11	3.4 (0.8)	3.0 (0.6)	3.8 (0.7)	3.1 (0.8)	3.0 (0.7)	3.9 (0.7)	3.9 (0.7)	3.3 (0.7)	3.8 (0.7)	4.1 (0.5)	2.7 (1.0)	3.4 (0.7)
12	3.0 (1.0)	2.6 (0.5)	3.1 (0.7)	3.5 (0.7)	2.7 (0.6)	3.4 (0.7)	3.5 (0.8)	3.2 (0.5)	3.2 (0.6)	3.6 (0.6)	3.0 (0.7)	3.2 (0.6)
13	3.3 (0.7)	3.1 (0.6)	3.5 (0.7)	3.5 (0.7)	2.9 (0.7)	4.2 (0.8)	4.0 (0.7)	3.4 (0.8)	3.8 (0.7)	3.9 (0.7)	3.3 (0.7)	3.6 (0.6)
14	3.8 (0.8)	3.4 (0.9)	4.0 (0.7)	4.0 (0.7)	3.4 (0.9)	4.3 (0.8)	4.4 (0.6)	3.7 (0.8)	4.0 (0.6)	4.1 (0.6)	3.4 (1.1)	3.9 (0.6)
15	3.4 (0.6)	2.7 (0.6)	3.2 (0.7)	3.3 (0.7)	3.0 (0.6)	3.8 (0.8)	4.0 (0.7)	3.4 (0.7)	3.6 (0.6)	3.9 (0.6)	3.3 (0.7)	3.5 (0.5)
16	3.5 (0.6)	2.8 (0.6)	3.7 (0.7)	3.8 (0.6)	3.2 (0.6)	4.0 (0.6)	4.3 (0.5)	3.6 (0.7)	4.2 (0.5)	4.1 (0.5)	3.2 (0.8)	3.7 (0.5)
17	3.6 (0.6)	3.2 (0.7)	3.8 (0.7)	3.9 (0.6)	3.2 (0.9)	4.4 (0.5)	4.4 (0.6)	3.6 (0.8)	4.1 (0.7)	4.5 (0.5)	3.7 (0.9)	4.1 (0.5)
18	3.0 (0.6)	2.8 (0.6)	3.1 (0.9)	3.4 (0.6)	2.9 (0.8)	3.5 (0.9)	3.6 (0.9)	2.9 (0.9)	3.3 (0.9)	3.4 (0.7)	2.7 (0.9)	3.1 (0.8)
19	3.1 (0.8)	3.0 (0.7)	3.4 (0.6)	3.1 (0.9)	3.0 (0.7)	4.0 (1.0)	4.0 (0.9)	3.0 (0.8)	3.4 (0.9)	4.0 (0.7)	3.0 (1.0)	3.5 (0.6)
20	2.9 (0.9)	2.5 (0.7)	3.4 (0.9)	2.7 (0.7)	2.2 (0.9)	3.2 (0.9)	3.4 (0.8)	2.7 (1.2)	3.2 (0.8)	3.5 (0.7)	2.9 (0.7)	3.0 (0.7)
21	3.4 (0.8)	2.7 (0.6)	3.5 (0.8)	3.3 (0.7)	3.0 (0.7)	3.6 (0.8)	3.7 (0.7)	3.1 (0.9)	3.6 (0.9)	3.5 (0.7)	2.6 (0.9)	3.2 (0.7)
22	3.2 (0.9)	2.9 (0.7)	3.6 (0.6)	3.8 (0.6)	3.2 (0.8)	4.2 (0.6)	4.3 (0.6)	3.8 (0.7)	3.9 (0.7)	4.2 (0.5)	3.8 (0.7)	3.7 (0.6)
23	3.6 (0.6)	3.2 (0.6)	4.0 (0.7)	3.9 (0.6)	3.5 (0.7)	4.6 (0.4)	4.5 (0.5)	3.9 (0.7)	4.2 (0.6)	4.2 (0.6)	3.7 (0.6)	3.8 (0.4)
24	3.5 (0.7)	3.0 (0.6)	3.5 (0.7)	3.7 (0.5)	3.1 (0.9)	4.0 (0.8)	4.2 (0.7)	3.6 (0.9)	3.9 (0.7)	4.1 (0.7)	3.5 (0.8)	3.6 (0.6)
25	3.8 (0.6)	3.1 (0.6)	3.7 (0.5)	3.7 (0.4)	3.5 (0.6)	4.3 (0.5)	4.3 (0.5)	3.8 (0.7)	4.1 (0.3)	4.3 (0.5)	3.8 (0.6)	3.8 (0.4)
Number of NHs scoring ≤3 for the dimension	5	16	0	1	12	0	0	4	0	0	11	2

Dimensions: 1. Teamwork, 2. Staffing, 3. Compliance with procedures, 4. Training and skills, 5. Non-punitive response to mistakes, 6. Handoff, 7. Feedback and communications about incidents, 8. Communication openness, 9. Supervisor Expectations and Actions Promoting Resident Safety, 10. Overall perceptions of resident safety, 11. Management Support for Resident Safety, 12. Organizational learning.
